# Serum neuroactive metabolites of the tryptophan pathway in patients with acute phase of affective disorders

**DOI:** 10.3389/fpsyt.2024.1357293

**Published:** 2024-04-12

**Authors:** Yanli Li, Leilei Wang, Junchao Huang, Ping Zhang, Yanfang Zhou, Jinghui Tong, Wenjin Chen, Mengzhuang Gou, Baopeng Tian, Wei Li, Xingguang Luo, Li Tian, L. Elliot Hong, Chiang-Shan R. Li, Yunlong Tan

**Affiliations:** ^1^Peking University HuiLongGuan Clinical Medical School, Beijing Huilongguan, Hospital, Beijing, China; ^2^Department of Psychiatry, Yale University School of Medicine, New Haven, CT, United States; ^3^Institute of Biomedicine and Translational Medicine, Department of Physiology, Faculty of Medicine, University of Tartu, Tartu, Estonia; ^4^Louis A. Faillace Department of Psychiatry and Behavioral Sciences at McGovern Medical School, The University of Texas Health Science Center at Houston, Houston, TX, United States

**Keywords:** bipolar disorder, major depressive disorder, tryptophan metabolism, kynurenine pathway, 5-hydroxytryptamine pathway

## Abstract

**Background:**

Many studies showed disrupted tryptophan metabolism in patients with affective disorders. The aims of this study were to explore the differences in the metabolites of tryptophan pathway (TP) and the relationships between TP metabolites and clinical symptoms, therapeutic effect in patients with bipolar disorder with acute manic episode (BD-M), depressive episode (BD-D) and major depressive disorder (MDD).

**Methods:**

Patients with BD-M (n=52) and BD-D (n=39), MDD (n=48) and healthy controls (HCs, n=49) were enrolled. The serum neuroactive metabolites levels of the TP were measured by liquid chromatography-tandem mass spectrometry. Hamilton Depression Scale-17 item (HAMD-17) and Young Mania Rating Scale (YMRS) were used to evaluate depressive and manic symptoms at baseline and after 8 weeks of antidepressants, mood stabilizers, some also received antipsychotic medication.

**Results:**

The levels of tryptophan (TRP) and kynurenic acid (KYNA) were significantly lower and the ratios of tryptophan/kynurenine (TRP/KYN), 5-hydroxytryptamine/tryptophan (5-HT/TRP), quinolinic acid/kynurenic acid (QUIN/KYNA) were higher in BD-M, BD-D, MDD vs. HC. The levels of QUIN and the ratios of QUIN/KYNA were higher in BD-M than in BD-D, MDD, and HCs. The 5-hydroxyindoleacetic acid (5-HIAA) levels of patients with MDD were significantly higher than those in BD-M and BD-D. Binary logistic regression analysis showed the lower peripheral KYNA, the higher the QUIN level, and the higher the risk of BD-M; the lower peripheral KYNA and the higher KYN/TRP and 5-HT/TRP, the higher the risk of BD-D; and the lower the peripheral KYNA level and the higher the KYN/TRP and 5-HT/TRP, the higher the risk of MDD. Correlation analysis, showing a significant association between tryptophan metabolites and improvement of clinical symptoms, especially depression symptoms.

**Conclusions:**

Patients with affective disorders had abnormal tryptophan metabolism, which involved in 5-HT and kynurenine pathway (KP) sub-pathway. Tryptophan metabolites might be potential biomarkers for affective disorders and some metabolites have been associated with remission of depressive symptoms.

## Highlights

- The levels of QUIN and QUIN/KYNA QUIN/KYNA were higher in patients with BD-M than those in patients with BD-Dand MDD and HCs.
- 5-HIAA levels of patients with MDD were significantly higher than those in patients with BD-M and BD-D.
- Binary logistic regression analysis showed tryptophan metabolites might be potential biomarkers for affective disorders but are not associated with the severity of clinical symptoms.

## 1 Introduction

Bipolar disorder (BD) and major depressive disorder (MDD) are the most common affective disorders. In China, the latest epidemiological study showed that the lifetime prevalence of MDD and BD was 6.8% and 0.6%, respectively (1). Because the onset of BD mainly manifests as depressive episodes, patients with BD are often misdiagnosed as MDD (2). About 69% of patients with BD were misdiagnosed as unipolar depression (UD) at onset, and it took 5 to 10 years before they were diagnosed as BD (3). Cross-sectional and symptomatological assessments are less conducive to distinguishing the two affective disorders, resulting in treatment delay. Therefore, studies on the etiological markers of BD and MDD are critical to the identification of the two diseases.

**Figure 1:**
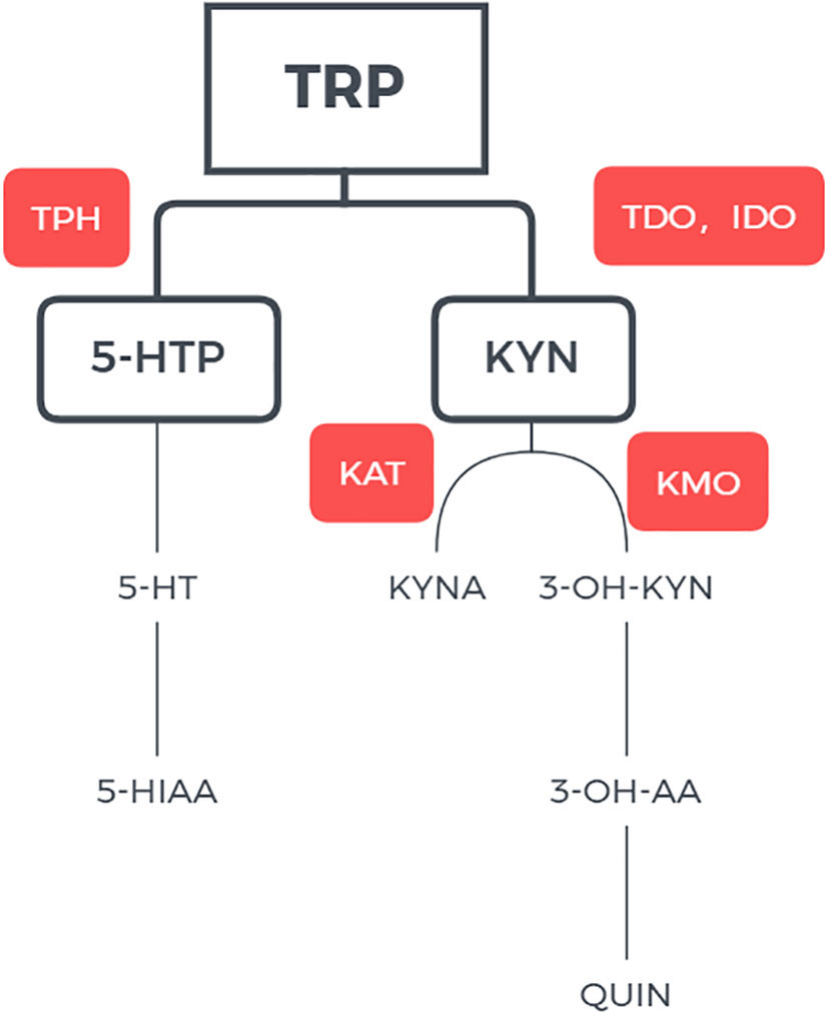
Pathway of tryptophan metabolisms. TRP tryptophan; IDO indoleamine 2,3-dioxygenase; TDO tryptophan 2,3-dixoygenase; KYN kynurenine; KAT kynurenine aminotransferase; KMO kynurenine-3-monoxygenase; 3-OH-KYN 3-hydroxykynurenine; 3-OH-AA 3-hydroxyanthranilic acid; QUIN quinolinic acid; QPRT quinolinate phosphoribosyltransferase; NAD+ nicotinamide adenine dinucleotide.

Abnormal tryptophan and serotonin (5-hydroxytryptamine or 5-HT) metabolism has been implicated in the pathophysiology of affective disorders (4). An essential amino acid, tryptophan is metabolized via two main pathways (5). Through the kynurenine (KYN) pathway (KP), 95% of tryptophan is metabolized to kynurenine KYN with the participation of two rate-limiting enzymes, tryptophan -2,3- dioxygenase (TDO) and indoleamine -2,3- dioxygenase (IDO). This pathway regulates the level of tryptophan (6). Kynurenic acid (KYNA) and quinolinic acid (QUIN) as produced by the metabolism have neuroprotective and neurotoxic effects, respectively, and the balance between the two metabolites plays important role in glutamatergic neurotransmission (7, 8). The remaining 5% of tryptophan is metabolized into 5-hydroxytryptophan (5-HTP) under the action of tryptophan hydroxylase, and 5-HTP is further metabolized into serotonin or 5-HT; under the action of aromatic L-amino acid decarboxylase. Serotonin can be further converted to melatonin or metabolized to 5-hydroxyindoleacetic acid (5-HIAA) and excreted in urine (**Figure 1**) (9).

The serotonin hypothesis suggests that depression is related to central serotonin dysfunction and deficiency (10). However, the findings on serotonin levels in depression are less than consistent. For instance, although some studies found lower 5-HT levels in plasma, serum, and platelets in patients with MDD (11–13), others reported otherwise (14, 15). In addition, some studies reported lower levels of 5-HIAA (16, 17) but others noted no significant reduction in 5-HIAA levels (18) in the cerebrospinal fluid (CSF) of patients with depression. Other studies noted no significant differences in central or peripheral 5-HIAA levels between depression patients and healthy controls (19, 20). For patients with BD as compared with healthy controls, although the serum 5-HT levels were lower (21), the concentration of 5-HT in the postmortem brain did not appear to be different (22). Likewise, studies have reported lower (23), indistinguishable (24), and higher CSF 5-HIAA levels in BD patients (25).

In a revised serotonin hypothesis of depression, depression is associated with lower plasma tryptophan and higher harmful tryptophan catabolites (TRYCATs) synthesis as a result of systemic inflammation (26). On the other hand, a meta-analysis showed lower levels of KYN and KYNA but not neurotoxic QUIN in patients with depression relative to healthy controls (27). It has even been shown that while TRYCATs are associated with depressive and anxiety symptoms and inflammatory states, all TRYCATs show no significant changes, in MDD vs. controls (28).

At present, there are few studies on the difference of tryptophan metabolites in acute MDD, BD-D, and BD-M patients, and the results are inconsistent. Studies on MDD and bipolar depression found no difference in peripheral TRP, KYN, KYNA, QUIN, KYNA/QUIN, KYNA/TRP, KYNA/KYN (21). Compared with healthy controls, patients with BD showed lower levels of TRP, KYN and KYNA during both manic and depressive episodes, but the levels of other metabolites did not appear to be altered in a consistent manner (29–31). Meta-analysis showed that patients with bipolar disorder had greatest reductions peripheral TRP levels during manic episodes and significantly lower KYNA levels during depressive episodes (32). With these discrepancies in extant findings, we compared the levels of TRP metabolites (5-HT and KP) for bipolar disorder in depressive episode (BD-D), manic episode (BD-M), and MDD. We explored the levels of different tryptophan metabolites as potential biomarkers for these affective disorders. We also investigated the predictive effect of tryptophan metabolites on the acute onset of affective disorders.

## 2 Methods

### 2.1 Participants

A total of 139 patients with BD-M (n=52), and BD-D (n=39) and MDD (n=48) from the Beijing Huilongguan Hospital were enrolled. The inclusion criteria were as follows: 1) age 18 to 60 years; 2) meeting the diagnostic criteria of BD-M, BD-D or MDD according to the Diagnostic and Statistical Manual of Mental Disorders, 5th Edition (DSM-5); 3) acute clinical episode; and 4) for BD-D and MDD, Hamilton Depression Scale-17 (HAMD-17) score ≥17; 5) for BD-M, Young Manic Rating Scale (YMRS) score ≥13. The exclusion criteria were as follows: 1) comorbid intellectual disability; 2) serious physical or brain organic diseases; 3) substance (except nicotine) use disorders; 4) comorbid Axis I disorders; and 5) pregnancy or lactation. Forty-nine healthy controls (HCs) matched by gender, age, and education were recruited from the local communities through advertising. This study was approved by the Medical Ethics Committee of Beijing Huilongguan Hospital. All participants have signed written informed consent prior to the study.

### 2.2 Blood sampling and assessment of biological indicators

All blood samples were collected at 7:00 a.m.- 8:00 a.m. after overnight (12h) fasting. 5 ml of cubital vein blood was taken and, after centrifugation and separation, immediately stored in -80°C refrigerator for testing. The concentrations of TRP, KYNA, KYN, QUIN, 5-HT, 5-HIAA were determined following standard procedures with liquid chromatography tandem mass spectrometry. Detailed steps are provided in the **Supplementary Materials 3**.

### 2.3 Clinical assessment

We assessed depressive symptom severity of patients with BD-D and MDD at baseline and 8 weeks after treatment by the HAMD. We used the 17-item version and the total score ranged from 0 to 54, 0 to 7 points without depression; 8-16 were classified as mild depression; 17-23 were classified as moderate depression; ≥ 24 is classified as major depression. The findings that a cut-off point of 17 on the HAMD may ensure a degree of severity of the depression, with higher scores indicating more severe depressive symptoms (33, 34). We used YMRS to evaluate the severity of manic symptoms at baseline and 8 weeks after treatment in patients with BD. The scale consists of 11 items and the total score ranges from 0 to 60, where 0 to 5 points: normal; 6-12 points: light; Scores 13-19: moderate; 20-29 points: severe; 30 and above: extremely heavy. In some studies, A total score ≥13 represents a potential case of mania or hypomania, while ≥21 indicates a probable case of mania or hypomania, with higher scores indicating more severe manic symptoms (35, 36). According to Seline van den Ameele et al. et al. ‘s research on tryptophan metabolites in bipolar disorder, we collected patients with YRMS or HAMD scores above moderate, so the cut-offs for YRMS≥13, HAMD≥17 (31).

Further, we employed the HAMD-17 and YMRS score reduction ratio to evaluate the therapeutic effect: reduction ratio = (baseline score - score at 8 weeks)/baseline score ×100%.

### 2.4 Statistics and data analyses

For demographic data, chi-square test was used to examine differences in gender composition among groups. One-way ANOVA was used to compare group differences in age, education level, and age of onset. The differences in the number of manic episodes between BD-M and BD-D were compared with Mann-Whitney U test. The number of depressive episodes was compared across groups by the Kruskal-Wallis test.

The ratios of KYN to TRP (KYN/TRP), QUIN to KYNA (QUIN/KYNA) and 5-HT to TRP (5-TH/TRP) were used to evaluate the conversion rate. Multivariate covariance analysis followed by post-hoc Scheffé tests to compare the differences in tryptophan and kynurenine metabolites among groups. Binary logistic regression was used to investigate the predictive effects of each tryptophan and kynurenine metabolite on BD-M, BD-D or MDD. Partial correlation analysis was performed to investigate the relationship between each tryptophan and kynurenine metabolites and baseline score and reduction ratio for HAMD and YRMS. There was no correlation between age or educational level and each tryptophan or kynurenine metabolite (*r’s*=0.02-0.13, *p’s*<0.05; *r’s*=0.01-0.09, *p’s*<0.05)*. T*-tests showed that the peripheral concentrations of TRP and KYN were higher in males than in females (*t*=3.57, *p<*0.001; *t*=2.95, *p*=0.004), so gender was taken as a covariate in multivariate covariance analysis, partial correlation analysis, and binary logistic regression. Bonferroni correction for 9 comparisons (9 KP metabolites), resulting in a threshold p-value of 0.006.

## 3 Results

### 3.1 Demographic and clinical features

There were no differences in gender composition, age, or education among the four groups. The age of onset of BD was significantly younger than that of MDD. BD-M had significantly more manic episodes than BD-D. Compared to BD-M and BD-D, MDD had significantly more severe depressive symptoms (**Table 1**).

**Table 1:** Demographics and clinical characteristics.

Variables	BD-M	BD-D	MDD	HC	χ^2/^F/Z/H	*p*	Multiple comparison
	(*N* = 52)	(*N* = 39)	(*N* = 48)	(*N* = 49)			
Sex Male Female							
	25 (48.1%)	16 (41.0%)	23 (47.9%)	24 (49.0%)	0.64	0.879	–
	27 (51.9%)	23 (59.0%)	25 (52.1%)	25 (51.0%)			–
Age, year	38.25 (11.03)	39.08 (9.25)	37.10 (10.27)	39.24 (11.47)	0.40	0.754	–
Education, year	11.90 (2.12)	11.64 (2.71)	12.02 (2.29)	12.63 (2.31)	1.47	0.244	–
Age at onset, year	25.87 (9.38)	30.08 (9.08)	29.19 (7.66)	–	3.06	0.050	BD-M<BD-D
Number of mania episodes^a)^	3.17 (1.79)	1.87 (1.13)	–	–	-3.74	<0.001	
Number of depressive episodes ^b)^	1.35 (1.44)	1.72 (0.65)	3.00 (1.14)	–	46.32	<0.001	BD-M, BD-D<MDD
Illness duration, day ^b)^	151.96 (118.01)	105.23 (81.98)	93.69 (97.04)	–	9.18	0.010	BD-M >MDD

BD-M, Bipolar disorder in manic episode; BD-D, Bipolar disorder in depressive episode; MDD, Major depression disorder; ^a)^ Mann-Whitney U test; ^b)^ Kruskal-Wallis test. "-" not applicable.

### 3.2 Differences in metabolites of tryptophan among different groups

**Figure 2:**
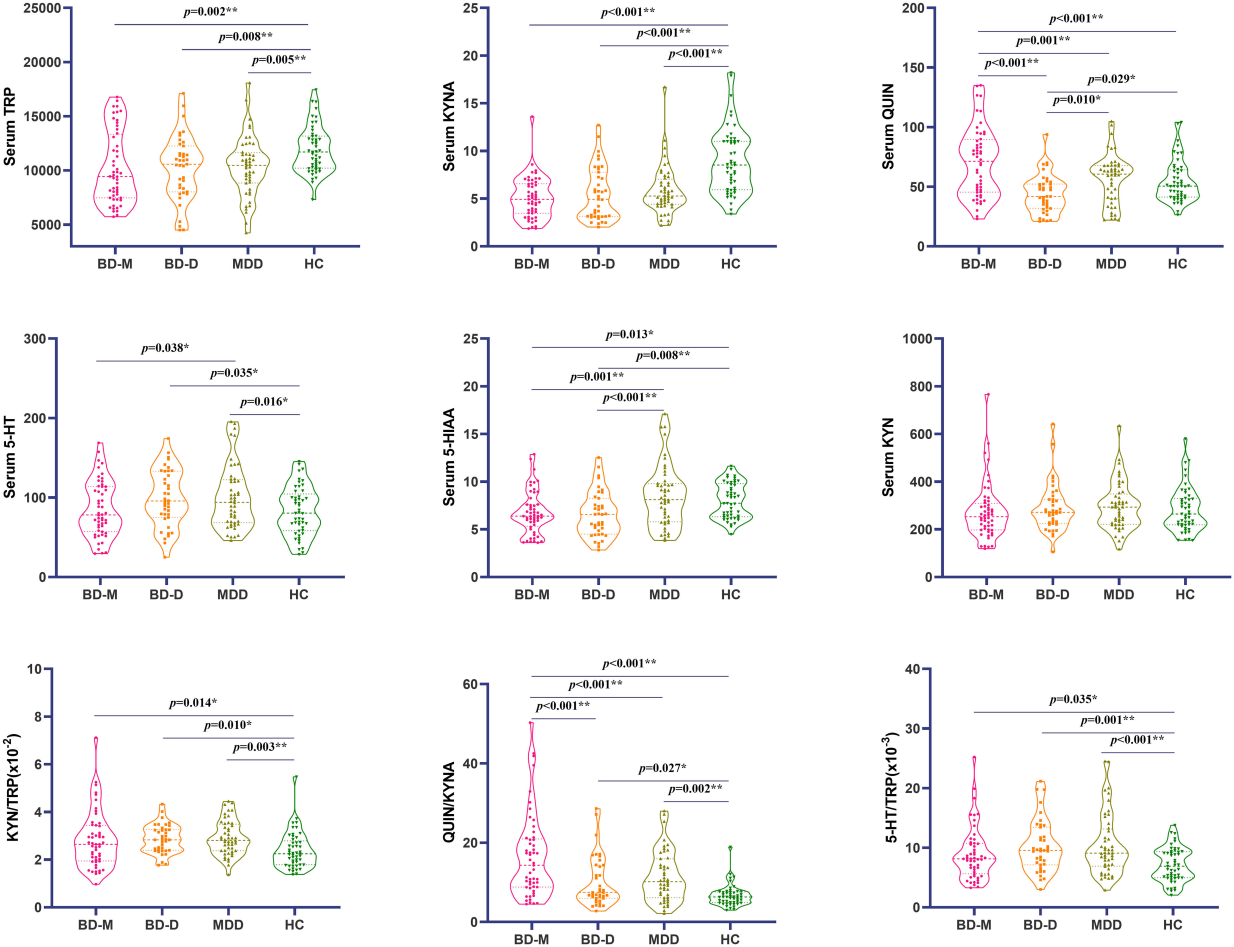
Comparison of metabolites of tryptophan and kynurenine in 4 groups using (mean ± standard deviation). *p < 0.01; **p < 0.009. BD-M, bipolar disorder in manic episode; BD-D, bipolar disorder in depressive episode; MDD, Major depression disorder; HC, Health controls.

After Bonferroni correction, multivariable covariance analysis showed that peripheral TRP and KYNA levels were significantly lower in the as compared with HCs. KYN/TRP were significantly higher when MDD compared to HCs, and 5-HT/TRP ratios were significantly higher in the BD-D, MDD groups as compared with controls. There was no difference in pairwise comparisons between any two of the disease groups for TRP, KYNA, KYN/TRP and 5-HT/TRP. The QUIN levels and QUIN/KYNA ratio of patients with BD-M were significantly higher than those in BD-D, MDD and HCs. The 5-HIAA levels of MDD were significantly higher than BD-M and BD-D, and the 5-HIAA level of HCs was also higher than BD-D. There was no difference in 5-HT levels between the groups comparisons. (**Figure 2**; **Supplementary Tables 2**).

### 3.3 Tryptophan and kynurenine metabolites predictors of the diagnosis of BD-M, BD-D or MDD

**Figure 3:**
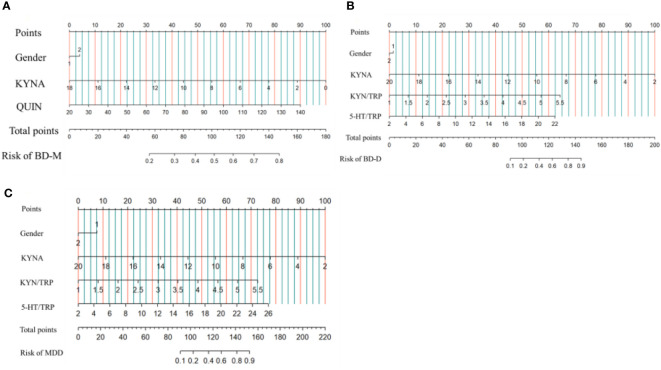
Risk factors of BD-M **(A)**, BD-D **(B)** and MDD **(C)** nomogram. (Code of sex, 1: male, 2: female) (To use the nomogram, an individual patient’s value is located on each variable axis, and a line is drawn upward to determine the number of points received for each variable value. The sum of these numbers is located on the Total Points axis, and a line is drawn downward to the Risk of BD-M, BD-D, MDD axes to determine the BD-M, BD-D, MDD risk).

Binary logistic regression analysis with BD-M/HCs as the dichotomous dependent variable and gender as covariate showed that the lower peripheral KYNA, the higher the QUIN level, and the higher the risk of BD-M. The same method was used to analyze the predictors of BD-D and MDD. The results showed that the lower peripheral KYNA, the higher KYN/TRP and 5-HT/TRP, the higher the risk of BD-D. The lower the peripheral KYNA level and the higher the KYN/TRP and 5-HT/TRP, the higher the risk of MDD. After bonferroni correction, the prediction effect of 5-HIAA on BD-D, QUIN on BD-D and 5-TH/TRP on MDD was not significant. The prediction effect of combined indicators on BD-M, BD-D or MDD was represented by a normogram (**Table 2**, **Figure 3**).

**Table 2:** Influence factors of BD-M, BD-D or MDD by binary logistic regression analysis.

Group	Variable	B	SE	OR	95%CI	*p*
BD-M vs HC	KYNA	-0.66	0.15	0.52	0.39∼0.69	<0.001
	QUIN	0.06	0.15	1.06	1.03∼1.09	<0.001
	5-HIAA	-0.33	0.14	0.72	0.54∼0.94	0.018
BD-D vs HC	KYNA	-0.41	0.13	0.66	0.55∼0.82	0.001
	QUIN	-0.07	0.03	0.94	0.89∼0.98	0.009
	KYN/TRP	1.62	0.53	5.05	1.81∼14.13	0.002
	5-TH/TRP	0.27	0.09	1.31	1.09∼1.57	0.004
MDD vs HC	KYNA	-0.40	0.10	0.67	0.55∼0.82	<0.001
	KYN/TRP	1.15	0.40	3.17	1.45∼6.93	0.004
	5-TH/TRP	0.23	0.08	1.26	1.07∼1.48	0.006

BD-M, bipolar disorder in manic episode; BD-D, bipolar disorder in depressive episode; MDD, Major depression disorder.

### 3.4 Relationship between each baseline tryptophan and kynurenine metabolite and clinical symptoms

Partial correlation analysis showed that neither tryptophan nor kynurenine metabolites were associated with HAMD (BD-D + MDD) and YMRS scores at baseline (BD-M only). KYN/TRP was negatively correlated with the reduction ratio of HAMD in BD-D + MDD. There was no correlation between tryptophan and kynurenine metabolites and reduction ration of YMRS in BD-M (**Table 3**).

**Table 3:** Relationship between each tryptophan and kynurenine metabolite and clinical symptoms.

	TRP	KYNA	QUIN	5-HT	5-HIAA	KYN	KYN/TRP (x10^-2^)	QUIN/KYNA (x10^-3^)	5-TH/TRP
	*r*(*p*)	*r*(*p*)	*r*(*p*)	*r*(*p*)	*r*(*p*)	*r*(*p*)	*r*(*p*)	*r*(*p*)	*r*(*p*)
Baseline score of HAMD	-0.05	0.14	0.08	-0.02	0.10	-0.20	-0.18	-0.02	0.04
	(0.669)	(0.212)	(0.499)	0.874	0.383	(0.071)	(0.099)	(0.876)	0.736
Reductive rate of HAMD	0.25	0.04	-0.15	0.28	-0.002	-0.03	**-0.38**	-0.01	0.06
	(0.022)	(0.697)	(0.164)	0.010	0.987	(0.790)	**(<0.001)**	(0.927)	0.603
Baseline score of YAMS	-0.06	-0.04	0.08	-0.07	0.16	0.16	0.27	0.14	-0.05
	(0.689)	(0.770)	(0.499)	0.601	0.257	(0.255)	(0.256)	(0.340)	0.712
Reductive rate of YAMS	-0.01	-0.07	-0.15	-0.03	0.17	-0.06	-0.02	0.34	0.02
	(0.944)	(0.654)	(0.164)	0.839	0.252	(0.681)	(0.890)	(0.017)	0.900

HAMD, Hamilton Depression Scale; YMRS, Young Mania Rating Scale. Bold values indicate that the P-value is still meaningful after Bonferroni correction.

## 4 Discussion

By firstly comparing the differences of tryptophan metabolite levels in patients with MDD, BD-D and BD-M, we found that relative to healthy controls, patients with MDD, BD-D and BD-M showed significantly reduced TRP levels, consistent with previous studies (32, 37). A meta-analysis of 10,912 participants in 101 studies also showed significantly reduced peripheral TRP levels in patients with MDD, BD, and schizophrenia (4). Our results showed that lower tryptophan level in patients with affective disorders might be related to the acceleration of tryptophan metabolism in the 5-HT and KP pathways. We observed that KYN/TRP of MDD patients were significantly higher, KYN/TRP frequently used as an index of IDO activity, Meta-analysis and previous studies have confirmed that patients with depression have higher KNN/TRP, which also indicates that increased neurotoxicity of downstream TRYCATs in MDD (38, 39).

### 4.1 Differences in 5-HT and 5-HIAA levels, 5-HT/TRP for patients with affective disorders

For the 5-HT branch, we found that patients with affective disorders had higher 5-HT/TRP during depressive episodes (both BD-D and MDD), although most previous studies have found lower 5-HT/TRP. when compared with healthy controls (11, 40). Our study found that 5-HT levels in patients with depressive episodes (both BD-D and MDD) in our study did not differ from those in healthy controls after Bonferroni correction. Most current studies have found a decrease in 5-HT levels during depressive episodes (14, 41, 42), some studies have found no change (43), but some studies have shown an increase (44, 45). The reasons for the difference in results are as follows: We considered the following: Firstly, the influences of 5-HT system on depressive episode are complex, involving 5-HT receptor (43, 46), 5-HT transporter and its genetic diversity (47), and the activity of related enzymes (48, 49). In addition, combined physical diseases, such as hypertension, coronary heart disease, diabetes, etc., can also affect 5-HT levels. The changes of 5-HT levels in the brain and periphery of patients with affective disorders warrant further study.

We found that patients with BD have lower levels of 5-HIAA during both depressive and manic episodes, as compared with MDD and healthy controls. However, there was no significant difference in 5-HIAA levels in depressive patients vs. controls, in accord with previous meta-analysis and cross-sectional studies (11, 20, 50). Together, the findings suggest that lower 5-HIAA levels may represent biochemical marker of BD. In support, previous studies of postmortem brain tissue in patients with BD have also found reduced 5-HIAA levels in the frontal and parietal lobes, and prospective studies have linked reduced CSF 5-HIAA levels to suicide attempts in patients with BD (22, 51).

### 4.2 Differences in kynurenine pathway for patients with affective disorders

Consistent with previous findings, we also observed that compared with healthy controls, patients with affective disorders have a higher neurotoxic ratio (QUIN/TRP) and a lower KYNA level, suggesting a “metabolic breakdown” toward the neurotoxic branch (52, 53). Neurodegeneration hypothesis hold that affective disorders, especially depression, reflect an imbalance between neuroprotective and neurotoxic metabolites in the KP pathway (48, 54). As the only endogenous NMDA receptor antagonist, KYNA has anti-oxidative and neuroprotective properties. KYNA regulates the release of neurotransmitters such as glutamic acid, dopamine (DA) and acetylcholine (ACh), mitigates the neurotoxicity of QUIN, and may reduce depression and mania symptoms (31, 55).

As a powerful endogenous excitatory toxin and NMDA-R agonist, QUIN participates in oxidative stress, cause excitotoxic injury, induce immune dysfunction and inflammation, and in the state of inflammation increase the risk of the peripheral QUIN entry in the brain, exacerbating neurotoxicity (56). However, in our study, QUIN levels were not altered in a consistent manner in patients with affective disorders, higher in BD-M, lower in BD-D, and no differences in MDD, relative to controls. In addition, QUIN showed the highest levels in BD-M, followed by MDD and BD-D. Whether this change is specific to different disease states remains unclear, but the finding suggests that bipolar manic episodes may be more closely related to the neurotoxic effects of QUIN. Further, our findings suggest that neurotoxicity caused by the imbalance between KYNA and QUIN metabolites may have a more dominant effect on depressive episodes.

Our results suggest that tryptophan metabolites represent potential biomarkers of BD-M, BD-D and MDD. These findings can be considered with previous studies highlighting the roles of peripheral KYN and 3-hydroxyl kynurenine and/or QUIN in the pathophysiology of affective disorders (52, 57).

### 4.3 Relationship between tryptophan and kynurenine metabolite and therapeutic effect

Our study showed that tryptophan and its metabolites were not significantly associated with the severity of depressive symptoms at baseline. Enko’s study also did not find a correlation between tryptophan metabolite levels and severity of depressive symptoms in MDD (58). Another study suggested that the influences of tryptophan metabolites and symptom severity depended on the onset age, but only for early onset depression (59).

Lower KYN/TRP levels were associated with higher reduction ratio of HAMD. The level of KYN/TRP represents the activity of IDO and TDO and reflects the inflammatory state. Low inflammatory level is associated with better antidepressant response, while high inflammatory level is associated with drug resistance (60, 61). In addition, the correlation between HAMD reduction rate and KYN/TRP may also be affected by age. Previous studies have found that the total score of HAMD is significantly negatively correlated with KYN/TRP in adult patients with depression, but there is no correlation in adolescent patients with depression (15).

Our study found that tryptophan metabolites did not seem to be closely related to mania symptoms and reduction ratio. It has also been shown previously that tryptophan metabolites have no effect on lamotrigine and valproate response, only high levels of TRP, KYN/TRP, QUIN were associated with poor response to lithium, but less than one fifth of our BD-D patients were taking lithium (7). Previous studies have also shown that tryptophan metabolic index (plasma tryptophan/amino acids) was related to YMRS reduction ratio (62). In general, there are few articles on the relationship between tryptophan metabolites and treatment effect prediction of bipolar manic episode, which is worthy of further discussion.

At present, the relationship between the abnormal tryptophan metabolic pathway and the pathogenesis of affective disorders was still controversial. Some studies suggested that abnormal tryptophan metabolism were associated with the disease state, and study found no difference in tryptophan metabolites levels in people with MDD in remission compared with HCs (38). Another meta-analysis showed that some tryptophan metabolite levels in patients with BD and MDD returned to normal with the remission of symptoms (63). However, most studies still believe that the occurrence of affective disorders is related to abnormal tryptophan metabolites. An 8-month follow-up of patients with BD found that KYNA levels were significantly lower than those of HCs, even after their symptoms had resolved (31). Cerebrospinal fluid results of MDD in remission showed that 5-HIAA levels and 5-HIAA/KYN in were significantly lower than those in HCs (64). In lipopolysaccharide-induced mouse models of depression, decreased levels of 5-HT in hippocampus and increased expression of IDO were observed. Knockout of IDO gene in mice or drug-induced inhibition of IDO expression significantly improved depressive behavior in mice (65). Therefore, the relationship between tryptophan metabolism and affective disorders is worth further investigation.

## 5 Limitations of the study and conclusion

We consider the following limitations. First, our small sample size may lead to an increased risk of type II errors. Second, the study focused on peripheral TRP metabolites, which were not representative of central levels, and changes in TRP metabolites were not measured after 8 weeks of treatment. Third, we didn’t consider the effect of inflammatory status on tryptophan metabolism. Fourth, we did not collect information on drugs for physical diseases, the types of drugs, and these drugs may also have an impact on tryptophan metabolism. Therefore, in future studies, we can use large sample prospective studies to quantify the changes of tryptophan and inflammation in cerebrospinal fluid to more comprehensively explore the changes of tryptophan metabolites in patients with affective disorders.

Together, our study found that patients with affective disorders have a dysfunctional tryptophan metabolic pathway, an imbalance in the KP pathway, a conversion to the neurotoxic branch. Tryptophan metabolites are helpful in the diagnosis of affective disorders and may be potential biomarkers for diagnosis.

## Data availability statement

The raw data supporting the conclusions of this article will be made available by the authors, without undue reservation.

## Ethics statement

The studies involving humans were approved by Medical Ethics Committee of Beijing Huilongguan Hospital. The studies were conducted in accordance with the local legislation and institutional requirements. Written informed consent for participation in this study was provided by the participants’ legal guardians/next of kin.

## Author contributions

YL: Writing – review & editing, Supervision, Project administration, Funding acquisition. LW: Writing – original draft, Methodology, Investigation, Formal analysis, Data curation. JH: Writing – review & editing, Methodology, Investigation, Data curation. PZ: Writing – review & editing, Resources, Methodology, Investigation. YZ: Writing – review & editing, Methodology, Investigation. JT: Writing – review & editing, Investigation, Data curation. WC: Writing – review & editing, Resources, Methodology, Investigation. MG: Writing – review & editing, Methodology, Investigation, Data curation. BT: Writing – review & editing, Supervision, Project administration. WL: Writing – review & editing, Supervision, Resources, Project administration. XL: Writing – review & editing, Supervision, Project administration. LT: Writing – review & editing, Supervision, Project administration. LH: Writing – review & editing, Project administration, Methodology, Conceptualization. CL: Writing – review & editing, Supervision, Project administration, Methodology, Conceptualization. YT: Writing – review & editing, Writing – original draft, Supervision, Project administration, Conceptualization.
