# Supplementary material for: Serum neuroactive metabolites of the tryptophan pathway in patients with acute phase of affective disorders
Source: Front Psychiatry. 2024 Apr 12;15:1357293. doi: 10.3389/fpsyt.2024.1357293 (PMC11046465; doi:10.3389/fpsyt.2024.1357293)
Supplement: Supplementary file 1 [file Table_1.docx]

**Treatments were used for patients**

The use of antipsychotics and mood stabilizers in the BD-M group was as follows: olanzapine + valproate (7), olanzapine + quetiapine (2), olanzapine (2); olanzapine + clozapine (1), valproate + quetiapine (23), olanzapine + lithium carbonate (1), lithium carbonate + clozapine (1), quetiapine (1), lithium carbonate + quetiapine (4), venlafaxine + valproate (1), ziprasidone + lithium carbonate (1). We converted each antipsychotic dose to chlorpromazine equivalent antipsychotic dosage. Chlorpromazine equivalent antipsychotic dosage (CPZ) (Andreasen, Pressler, Nopoulos, Miller, & Ho, 2010; Gardner et al., 2010; Tachibana et al., 2016) in BD-M patients was 438.99 ± 527.64mg/d (mean ± SD). The average dose of valproate was 565.38 ± 491.85mg/d (mean ± SD). The average dose of lithium carbonate was 95.19 ± 288.04mg/d(mean ± SD).

The use of antipsychotics, mood stabilizers and antidepressant in the BD-D group was as follows: aripiprazole + duloxetine (4), amisulapride + votioxetine (1), olanzapine + votioxetine (1), valproate + amfebutamone (1), amfebutamone + lithium carbonate (1), amfebutamone + quetiapine (1), amfebutamone (1), sertraline +quetiapine (3), valproate + sertraline (2), sertraline + sulpiride (1), sertraline+ lithium carbonate (1), sertraline + paliperidone (1), citalopram + quetiapine (3), citalopram (1), citalopram + lithium carbonate (1), citalopram + olanzapine (1), citalopram + risperidone (2); escitalopram + paliperidone (1), escitalopram + olanzapine (1), escitalopram + quetiapine (2), escitalopram + valproate (1), venlafaxine + olanzapine (2), venlafaxine + risperidone (2), venlafaxine + quetiapine (1). We converted each drug to a citalopram equivalent, and the citalopram equivalent in BD-D was 31.67 ± 14.43mg/d (mean ± SD). Chlorpromazine equivalent antipsychotic dosage in BD-D patients was 200.87 ± 185.13mg/d (mean ± SD). The average dose of lithium carbonate was 47.37 ± 163.97mg/d(mean ± SD).

The use of antipsychotics, mood stabilizers and antidepressant in the MDD group was as follows: agomelatine (1), fluoxetine (2), mirtazapine + amisulapride (1), mirtazapine (2), trazodone hydrochloride (1), fluvoxamine + boonanserin (1), fluvoxamine (1), paroxetine (3); Duloxetine + quetiapine (2)， duloxetine (5); votioxetine (2); sertraline + quetiapine (1); sertraline + olanzapine (1), sertraline (3); citalopram + agomelatine (1), citalopram (5), escitalopram (5), venlafaxine + aripiprazole (1), venlafaxine + mirtazapine (1), venlafaxine plus quetiapine (1), venlafaxine (6); mianserin (1). We converted each drug to a citalopram equivalent, and the citalopram equivalent in MDD was 43.52 ± 15.90mg/d (mean ± SD)
