# Supplementary material for: Serum neuroactive metabolites of the tryptophan pathway in patients with acute phase of affective disorders
Source: Front Psychiatry. 2024 Apr 12;15:1357293. doi: 10.3389/fpsyt.2024.1357293 (PMC11046465; doi:10.3389/fpsyt.2024.1357293)
Supplement: Supplementary file 2 [file Table_2.docx]

**Supplementary Tables 2.** Comparison of metabolites of tryptophan and kynurenine in 4 groups using (mean ± standard deviation).

| Variables | BD-M | BD-D | MDD | HC | *χ^2^ / F / Z / H* | *p* | Multiple  comparison |
| --- | --- | --- | --- | --- | --- | --- | --- |
|  | (*N* = 52) | (*N* = 39) | (*N* = 48) | (*N* = 52) |  |  |  |
| TRP | 10230.34(3322.93) | 10242.76(2981.06) | 10350.54(2744.70) | 11937.49(2170.36) | 6.50 | <0.001 | BD-M,BD-D,MDD<HC |
| KYNA | 5.07(2.16) | 5.48(2.78) | 5.86(2.55) | 8.95(3.46) | 19.76 | <0.001 | BD-M,BD-D,MDD<HC |
| QUIN | 70.49(29.30) | 43.86(15.99) | 56.25(20.72) | 54.35(17.59) | 11.34 | <0.001 | BD-D,MDD,HCs<BD-M |
| 5-HT | 85.48(34.88) | 98.78(35.60) | 100.31(40.08) | 82.80(30.66) | 3.05 | 0.030 | - |
| 5-HIAA | 6.75(2.24) | 6.57(2.38) | 8.48(3.23) | 8.00(1.85) | 6.54 | <0.001 | BD-M, BD-D<MDD  BD-D<HCs |
| KYN | 272.96(118.70) | 289.96(101.61) | 296.95(99.32) | 282.66(94.07) | 0.55 | 0.647 | - |
| KYN/TRP(×10^-2^) | 2.82(1.19) | 2.87(0.59) | 2.91(0.73) | 2.39(0.76) | 3.77 | 0.012 | - |
| QUIN/KYNA | 16.71(10.61) | 10.02(6.23) | 11.32(6.35) | 6.65(2.71) | 17.05 | <0.001 | BD-D,MDD,HC<BD-M;  MDD>HCs |
| 5-TH/TRP(×10^-3^) | 8.95(4.46) | 10.25(4.39) | 10.44(5.15) | 7.14(2.86) | 5.83 | 0.001 | BD-M,BD-D,MDD>HC |
