# Supplementary material for: Serum neuroactive metabolites of the tryptophan pathway in patients with acute phase of affective disorders
Source: Front Psychiatry. 2024 Apr 12;15:1357293. doi: 10.3389/fpsyt.2024.1357293 (PMC11046465; doi:10.3389/fpsyt.2024.1357293)
Supplement: Supplementary file 3 [file Table_3.docx]

**LC-MS analysis:** (1) Sample pre-treatment: 200ul of serum was taken into 1.5ml EP tube, 600ul of internal standard precipitator was added, vortex and shake for 3min, then put into centrifuge, centrifuged at 15000rpm for 10min at 4℃, 700W of supernatant was taken into another EP tube, and then dried with nitrogen at 85℃. Add 1500 pure water to redissolve, and finally take 120ul supernatant by centrifugal again at high speed for testing. (2) Chromatographic conditions :Acquity UPLC-Class TQ ultra-high performance liquid chromatograph (Waters BEHC18 2.1x50mm 1.7um). Mobile phase: A: water (containing 0.1% formic acid), B: acetonitrile (containing 0.1% formic acid); Gradient elution, analysis time 5min, sample size 5ul, flow rate 0.35ml/min. Gradient elution: 0-1min,1%B; 1-3min, 1%-60%B; (3) Mass spectrum conditions: Instrument name: Waters TQS. Ion source: ESI+; Monitoring mode: MRM; Ion source parameters: ESI; Spray voltage: 3.5KV; Cone-hole voltage :20V; Desolvent temperature: 500℃; Desolvent gas: 1000l/hr; Cone hole blowback: 150l/hr. (4) Data calculation: Chromatogram collection and integration of kynurenine metabolites were processed by Analyst software.
